# The Effects of Fermented* Laminaria japonica* on Short-Term Working Memory and Physical Fitness in the Elderly

**DOI:** 10.1155/2018/8109621

**Published:** 2018-06-12

**Authors:** Storm N. S. Reid, Je-kwang Ryu, Yunsook Kim, Byeong Hwan Jeon

**Affiliations:** ^1^Department of Physical Education, School of Sports and Health, Kyungsung University, Busan 48434, Republic of Korea; ^2^Institute for Cognitive Science, College of Humanities, Seoul National University, Seoul 08826, Republic of Korea; ^3^Marine Bio-Industry Development Center, Marine Bioprocesses Co., Ltd., Busan 46048, Republic of Korea

## Abstract

Considering the neuroprotective and antioxidant potential of fermented* Laminaria japonica* A. (FST), the purpose of the present study is to establish whether FST may be considered a viable, efficacious supplement that can be administered in later life to offset neurodegenerative conditions associated with aging. Forty senior subjects participated in a randomized, double-blind, and placebo-controlled study. Two groups were formed, one FST group (n = 32, 72.35 ± 5.54 yrs) and one placebo (CON) (n = 28, 74.57 ± 5.69 yrs), which received 1.5 g/day of FST for 6 weeks. Subjects were asked to abstain from any regular exercise. In order to analyze short-term memory, a variety of neuropsychological tests were implemented. Body composition, physical fitness evaluations, antioxidant function, and inflammatory markers were also included in the analyses pre- and posttest. We demonstrated that FST significantly improved neuropsychological test scores, including higher scores in the K-MMSE, numerical memory test, Raven test, and iconic memory, compared to the CON group. Shorter test trial times in the 6-minute walk test were observed in the FST group (*p*<0.001 and* p*<0.05, trials 1 and 2, respectively). FST also significantly increased antioxidant activity of GPx, GSR, and SOD, reduced the production of TBARS, and lowered 8-oxoDG levels. The present study highlights the potential widespread application of FST in protecting against the degenerative effects of aging on short-term memory and physical function. Neuropsychological evaluation indicates that FST may provide a protective mechanism against cognitive impairment associated with dementia. Neuromuscular integrity and physical function are typically compromised in aging and dementia patients; thus, whether by stimulation of muscle-related growth factors or an increase in serum BDNF, FST supplementation may act to preserve physical function in the elderly. The bioactive constituents of FST such as GABA and fucoidan acting to provide improvements in antioxidant activity following FST supplementation may protect against progressive degeneration purportedly caused by reactive oxygen species.

## 1. Introduction

The prevention of age-related degeneration and diseases, such as cancer, diabetes, liver disease, stroke, and neurodegeneration, continues to dominate medical research. These conditions can severely affect quality of life (QoL) and the ability to carry out activities of daily living (ADLs). Later life predisposes the aging population to debilitating neurodegeneration that often results in cognitive impairment and may develop into conditions such as dementia and Alzheimer's disease [1]. Dementia tends to manifest in the form of behavioral, emotional, and cognitive impairments, including disorientation, mood swings, confusion, and short-term memory loss [2], hampering one's ability to perform ADLs. The global incidence of dementia was reported to be 35.6 million in 2010, with this number expecting to nearly double by 2030 to 65.7 million and 115.4 million in 2050 [3]. Thus, preservation of neuronal health and integrity, in the face of senescence, must be a priority when aiming to improve late-stage quality of life.

Oxidative stress (OxS) plays a key role in multiple disease states and causes serious cell damage if the stress is massive or prolonged. It is well known that excessively high levels of reactive oxygen species (ROS) and reactive nitrogen species (RNS) cause damage to cellular proteins, membrane lipids, and nucleic acids. The involvement of OxS in age-associated cognitive impairment and the pathogenesis of dementia and Alzheimer's disease (AD) is also a widely supported theory [4–6]. Systemic OxS [7] and, more specifically, intracellular production of oxidants and prooxidants over a prolonged period stimulate a cascade of ROS and RNS formation which contributes to the dysfunction of the blood-brain barrier, lipid peroxidation, oxidation of proteins and nucleic acids, and damage to brain cells, even cell death [8]. Furthermore, Lovell and Markesbery [9] concluded that DNA oxidation, particularly to mitochondrial DNA (mtDNA), is an early detectable sign of mild cognitive impairment (MCI), and that over time our bodies become increasingly less capable of repairing this damage, potentially reducing the expression of vulnerable genes involved in memory, learning, and neuronal survival. This MCI phase, commonly preceding the onset of dementia, is also characterized by significantly elevated RNA and protein oxidation and lipid peroxidation in vulnerable regions of the brain, demonstrated in humans [9–11]. The diagnostic process in identifying MCI highlights the cognitive decline that coincides with the presence of OxS. Hence, the proposed implementation of diagnostic neuropsychological assessments [12] using instruments such as the memory assessment scales (MAS) [13], mini-mental state examination (MMSE) [14], and iconic memory and Raven tests ([15, 16], respectively) which aid in demonstrating a relationship between the decline in memory, language, attention, and visuospatial skills, respectively, in MCI likely associated with OxS. Similarly, the presence of OxS and depleted plasma antioxidant activity in patients with MCI [17, 18], taken together with the ability to induce neurological deficits, similar to those of AD, by creating an antioxidant vitamin deficiency [19], reversible in humans by vitamin supplementation [20], is further evidence for the causative role of OxS in the pathogenesis of neurodegenerative disease, but also the potential of nutritional interventions in treating such conditions.

Administration of functional foods that are readily available and much less likely to cause adverse side effects has shown promising results, particularly with the use of edible seaweeds. Of these,* Laminaria japonica *A., an age-old Pacific and Asian food resource, typically consumed for its high contents of dietary fiber, carbohydrates, minerals, and protein, has attracted growing interest for its bioactive potency and prophylactic activity in vivo [21, 22]. A plethora of amino acids have been found to occur in FST that are increased as a result of fermentation including alanine, valine, glycine, and leucine. Other key bioactive compounds found in* Laminaria japonica *A. include sulfated polysaccharides such as fucoidans and polyphenols which have been implicated in many of the following prophylactic effects of FST [23]. Early studies have demonstrated the antioxidant properties of* Laminaria japonica* A. [24] as well as its hypotensive effects [25], antimutagenic [26], antihyperlipidemic, and antiatherosclerotic activities, in vivo and in vitro [27]. More recently, studies have applied a specialized fermentation process to the sea tangle using* Lactobacillus brevis* BJ20 to enrich the gamma amino butyric acid (GABA) content eliciting significant antioxidant effects. These studies reported reduced diphenylpicrylhydrazyl (DPPH) levels, xanthine oxidase inhibition in vitro [23], increased superoxide radical scavenging, enhanced catalase, decreased malondialdehyde (MDA) levels [28], and decreased lipid peroxidation [29] following fermented sea tangle (FST) supplementation. GABA is believed to play a key role in the antioxidant effects of FST which may provide a range of health-promoting benefits in MCI. GABA administration has been shown to inhibit the formation of advanced lipoxidation end-products [30] and, more recently, decrease MDA concentration while increasing the activities of superoxide dismutase (SOD) and glutathione peroxidase (GPx) in the cerebral cortex and hippocampus, in vivo and in vitro [31], brain regions crucial to memory formation. We also demonstrated GABA-enriched FST supplementation in middle-aged women stimulates the release of muscle-related growth factors and increase serum brain-derived neurotrophic factor (BDNF) [32], known to provide protection for innervating motor neurons, stimulate protein synthesis [33, 34], and maintain neuromuscular integrity [32] in age-related muscular degeneration [35]. BDNF is also strongly implicated in the pathogenesis of dementia [36], AD [37], and Parkinson's disease [38].

Therefore, with the neuroprotective and antioxidant potential of FST, the purpose of the present study is to establish whether FST may be considered a viable, efficacious supplement that can be administered in later life to offset neurodegenerative conditions associated with aging.

## 2. Material and Methods

### 2.1. Participants

Sixty moderately active senior subjects participated in a randomized, double-blind, and placebo-controlled study in which the supplement company providers randomly assigned a number to opaque containers which were randomly distributed to subjects by the researchers. (Table 1). Two groups were formed, one FST group (n = 32, 72.35 ± 5.54 yrs) and one placebo (CON) (n = 28, 74.57 ± 5.69 yrs). The FST group ingested 1.5 g/day of FST for 6 weeks, while the placebo group consumed a sucrose pill. Subjects suffering from hypertension, diabetes, chronic degenerative disease (arthritis), cardiovascular disease, and/or obesity were excluded from the study. Participants were also excluded if they experienced an adverse reaction to supplementation prior to the experimental period or were unable to participate due to particular medication. All participants completed a written informed consent to partake in this study, which was approved by Seoul National University Ethics Committee (IRB no. 1508/001-005). We experienced participant dropout mainly due to participants not wanting to partake in certain procedures, namely, blood testing, which enabled us to assess the neuropsychological ability of all aforementioned participants; however, antioxidant activity and growth factor assessment was carried out on the remaining FST participants (n=20) and twenty placebo participants (n=20).

### 2.2. Preparation of FST by Lactobacillus brevis BJ20 and Placebo

**Figure 1:**
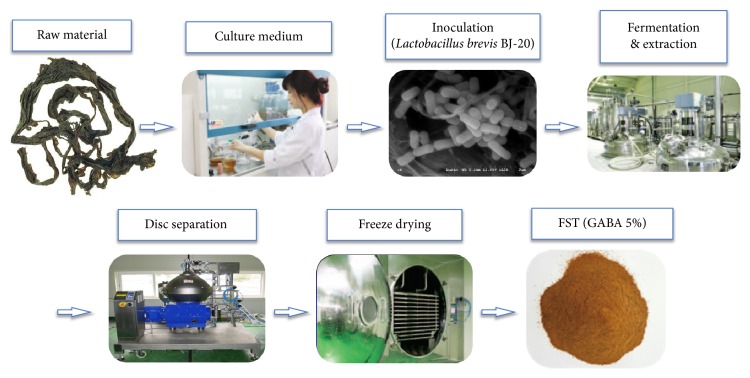
Preparation process of y-aminobutyric acid- (GABA-) enriched fermented sea tangle (FST) from raw sea tangle. Adapted from “effects of *γ*-aminobutyric acid-enriched fermented sea tangle (Laminaria japonica) on brain-derived neurotrophic factor-related muscle growth and lipolysis in middle-aged women,”* *Algae,* *vol. 31, no. 2, pp. 177, 2016. Adapted with permission.

FST was prepared following a recently modified procedure [32]. Sea tangle was added to water at a ratio of 1:15 (w/v) with the addition of yeast extract and glucose, based on the amount of sea tangle added to the mixture. After autoclaving at 121°C for 30 min, a sea tangle solution was obtained and the* Lactobacillus brevis* BJ20 (Accession No. KCTC 11377BP) culture broth was added to the solution at a concentration of 1.2% (v/v) (Table 2), which was mixed and incubated at 37°C for 2 days (Figure 1). A soft capsule was made for the clinical trial by mixing 250 mg fermented sea tangle, 61 mg lactose, 32 mg cellulose, 5 mg HPC, 30 mg SiO2, and 7 mg magnesium stearate. A placebo was made by mixing 311 mg lactose, 32 mg cellulose, 5 mg HPC, 30 mg SiO2, and 7 mg magnesium stearate.

During the preparation process, the GABA content had been controlled to range within 40~60 mg within 1,000 mg of FST. Based on high performance liquid chromatography analysis, it was confirmed that the mean content of GABA was 54.5 ± 0.071 mg/g^−1^ in the FST used in this study.

### 2.3. General Protocol

#### 2.3.1. Determination of Antioxidant Enzyme Activities

Serum SOD activity was determined using a commercially available kit, Cayman's Superoxide dismutase assay kit (Cayman Chemical Company, Ann Arbor, MI, USA). Tetrazolium salt was utilized to detect superoxide radicals generated by xanthine oxidase and hypoxanthine, which react to form a yellow formazan dye. The detection limit of the assay was 0.025-0.25 U/ml SOD. Inter- and intra-assay coefficients of variation were 3.7 and 3.2%, respectively. Absorbance was measured at a wavelength of 440-460 nm and expressed as U/ml.

Serum GPx activity was measured using the Cayman GPx Assay Kit (Cayman Chemical Company, Ann Arbor, MI, USA). The cosubstrate mixture contained NADPH, glutathione, and glutathione reductase, and the reaction was initiated by adding 20 *μ*l of cumene hydroperoxide. One unit of GPx was defined as the amount of enzyme that catalyzes the oxidation of 1.0 nmol of NADPH per minute at 25°C. Absorbance was measured at a wavelength of 340 nm and expressed as U/ml.

Serum GSR activity was measured using the commercially available Cayman Chemical Glutathione Reductase Assay Kit (Cayman Chemical Company, Ann Arbor, MI, USA), which measures the rate of NADPH oxidation. The reaction was initiated by adding 50 *μ*l of NADPH and accompanied by a rapid decrease in the absorbance at 340 nm. One unit of GSR activity was calculated as the amount of enzyme that catalyzes the oxidation of 1.0 nmol of NADPH per minute at 25°C

Serum TBARS content was determined with the OxiSelect™ TBARS Assay Kit, a commercial kit provided by Cell Biolabs Inc. (San Diego, CA, USA). The malondialdehyde containing samples were first reacted with TBA at 95°C. After a brief incubation, the malondialdehyde-protein adducts content in the serum was determined by comparison with a predetermined MDA standard curve. Spectophotometric measurement was used to calculate TBARS at 532 nm absorbance. Serum TBARS content was expressed as *μ*M.

Oxidative stress marker, 8-oxoDG, was measured using a competitive enzyme-linked immunosorbent assay kit from JaICA (Japan Institute for the Control of Aging, Shizuoka, Japan), following manufacturers' instructions. This test used a monoclonal antibody N45.1. Absorbance from the wells was measured at 450 nm with a microtiter plate reader.

#### 2.3.2. Growth-Related Factors

HGH concentration was determined using Immulite 2000, a commercially available kit from Siemend AG (Muenchen, Germany). A growth hormone releasing hormone + arginine stimulation rest calibrated with both IS 80/505 and IS 98/574 (GRH Growth Hormone-Recombinant 98/574-kit).

Insulin-like growth factor-1 (IGF-1) concentrations were measured using a solid-phase enzyme-labelled chemiluminescent immunometric assay on the Immunlite 2000 automated Immunoanalyzer (Siemens AG).

For measuring BDNF concentrations, a commercially available kit from R&D Systems, Inc. (Minneapolis, USA), was used. Diluted serum was used to determine the optical density of each sample well within 30 minutes at 450 nm.

#### 2.3.3. Neuropsychological Tests

*K-MMSE. *The MMSE is a brief cognitive status instrument that assesses orientation to time and place, registration, memory, attention and concentration, praxis, constructional and language capacity, and ability to follow commands [39]. “K” simply denotes a Korean translation of the aforementioned test. The unidimensionality of the MMSE can be assumed for this population sample [40]. Scoring of the items was binary in nature; meaning correct responses were coded as 0 and incorrect responses as 1. Scores were determined by summing the points assigned to each successfully completed task; the maximum score is 30. In order to indicate cognitive impairment a cutting point score of 23/24 was used. This demonstrates that none of the subjects in the current study were determined cognitively impaired.

*Numerical Memory Test. *Participants were asked to attempt to memorize a stimulant, in the form of a sequence of numbers, in order to assess short-term working memory.

*Raven's Standard Progressive Matrices. *Utilized as a nonverbal assessment tool measure participants' intelligence [41], each item of the Raven's test comprises a pattern of diagrammatic puzzles with one piece missing. Participants must choose the correct missing piece from a series of possible answers. There is a time limit of 47 minutes to complete the test.

*Flanker Test. *This test was used to measure frontal lobe function (e.g., executive function). Participants needed to suppress the autonomous response to surrounding distractors by rapidly determining the direction of a central target (arrow) while effectively inhibiting distracting information.

*Iconic Memory Test (Pre, Simultaneous, Post). *Participants were required to remember and report the sensory stimuli presented on a screen for a short time in order to assess sensory memory capacity. Three experimental conditions were used in this study: precue condition, indicator signal appears for 200 ms before the stimulus array; simultaneous condition, instructional signal appears simultaneously with the stimulus array; postcue condition, stimulation signal appears 200 ms after the stimulus array.

*Trail Making Test (TMT). *The procedure carried out was based on computer administered test previous utilized in the literature [42]. The computer display for the trial task consisted of 25 targets randomly scattered on a 10 x10 grid. Participants were directed to use the left, right, down, and up arrow keys on the keyboard to move a cursor through the sequence of targets as quickly and efficiently as possible. Each keystroke moved the cursor on the computer screen one space, but no record or trail of previous positions of the cursor was shown. The total time to move the cursor through the entire sequence of targets was recorded.

#### 2.3.4. Physical Function Measurements

*6-Minute Walk Test (6MW). *The 6MW test was used to measure the maximum distance that a person can walk in 6 minutes. This test is a submaximal aerobic capacity test that allows researchers to safely and simply assess physical function with the elderly and special populations [43, 44].

*Timed Up and Go Test (TUG). *This test was carried out by timing how long it takes for a subject to stand up from an arm chair, walk a distance of 3 m, turn, walk back to the chair, and sit down [43, 44]. TUG is used as an indicator of basic mobility skills.

## 3. Statistics

All statistical analyses were conducted with Statistical Package for Social Sciences (SPSS Windows version. 21.0; SPSS Inc., Chicago, IL., USA). Descriptive statistics (mean and standard deviation) were used to report on the measurements of each variable. In the pretest evaluation,* t*-tests were conducted to ensure the homogeneity of the FST group and the CON group. In order to verify the impact of FST supplementation, a* t*-test was conducted between groups. Statistical significance was set at *α* = 0.05.

## 4. Results

### 4.1. Neuropsychological Tests

Analysis of K-MMSE scores before and after supplementation demonstrated a significant effect of FST ingestion on cognitive function. The increases in scores found in the FST group were significantly different to the relatively unchanged scores in the CON group (p<0.05). The analysis of working memory function using a numerical memory test showed a significantly greater improvement in scores achieved by the FST group compared to the CON group (p<0.05). The Raven test was utilized to assess the visual and spatial reasoning of participants. Pre- and posttest scores indicate a significant improvement following 6 weeks of FST supplementation compared to a reduction in posttest scores in the CON group (p<0.001). The Flanker test was implemented to further evaluate cognitive function, specifically, information processing and selective attention. Analysis showed that there was a significant reduction in response time in all groups, but no statistically significant difference was observed between the changes in the FST group and CON group after the 6-week program (Table 3).

In order to analyze visual sensory memory, the iconic memory test was used. Results were reported under three separate conditions: precue, simultaneous cue, and postcue. Precue condition: both FST and CON groups demonstrated an increase in iconic memory test score times after the 6 weeks of supplementation. Although there was no significant difference between groups, the greater improvement following FST ingestion may indicate an enhanced ability to perceive the visual stimuli and perform the identification task. Simultaneous cue condition: a statistically significant difference was observed between the FST and CON group iconic memory pre- and posttest durations (p<0.05). Postcue condition: as previously observed in the simultaneous cue condition, both test times were improved following FST and CON group supplementation; however there was a significantly greater improvement in the FST group (p<0.05) (Table 3). This may indicate that FST has an enhancing effect on sensory memory maintenance ability following visual stimuli.

The trail-making task offered measurements pertaining to the speed and cognitive fluidity of the elderly population at hand. Following supplementation of FST and placebo administration, trail-making task time was reduced in both groups but without a significant difference between groups.

### 4.2. Body Composition and Health-Related Fitness

Analysis of body composition showed no difference in either the FST or CON group after 6 weeks of supplementation. On the other hand, significant changes were observed in measurements of physical fitness, as shown in Table 3. Both trials of the 6MW test time were reduced in the FST group and increased in the CON group, representing a significant difference between groups (6MW (1), p<0.001; 6MW (2), p<0.05). Both TUG test trial times were also improved in the FST group, compared to the longer test times observed in the CON group, though only significantly different in the TUG (1) test trial. These results suggest functional improvements elicited by ingestion of FST.

### 4.3. Antioxidant Function

**Figure 2:**
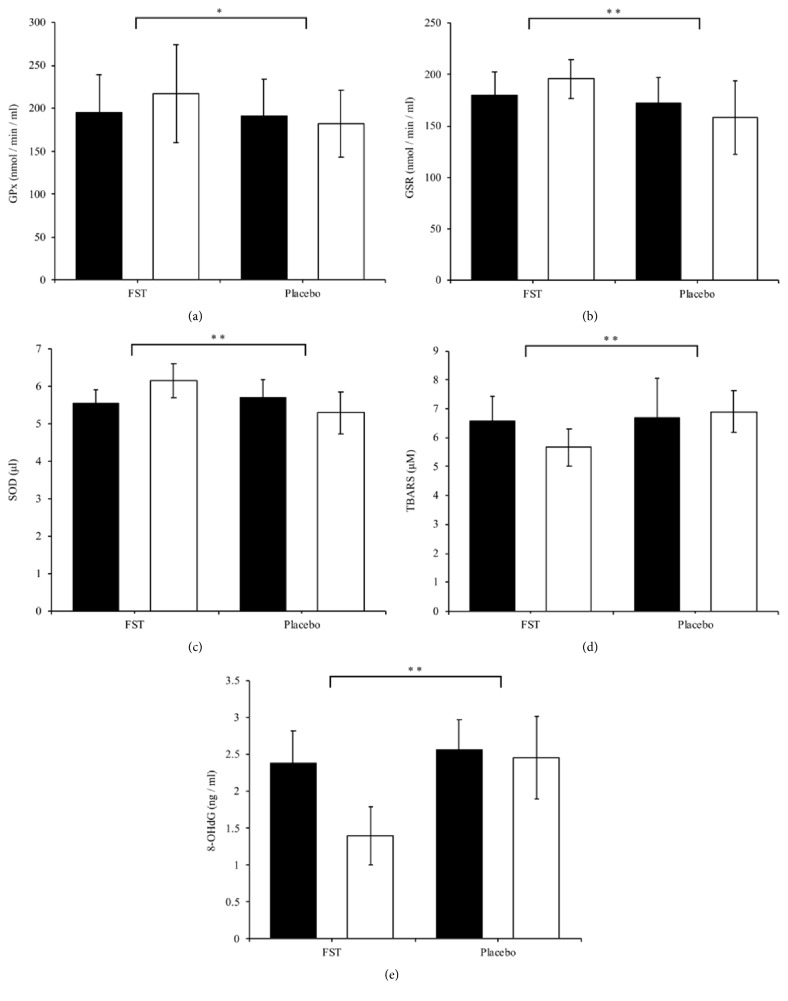
Serum (a) GPx, (b) GSR, (c) SOD, (d) TBARS, and (e) 8-oxoDG levels were determined in the FST and placebo group, pre- and postexperimental trial. Each bar represents the mean ± SD. Student's paired* t*-tests were carried out to determine significant differences (*∗*p < 0.05, *∗∗*p < 0.001) between the FST and placebo groups.

As shown in Figures 2(a), 2(b), and 2(c), antioxidant activities of GPx, GSR, and SOD were all increased in the FST group compared to the reduction in activity of these antioxidant molecules in the CON group. These differences between groups were significant (p < 0.05, p = 0.001 and p = 0.001, respectively). The aforementioned results imply that FST possess antioxidant properties that may contribute to bolstering the body's defense against oxidative stress by helping to maintain a “normal” redox balance.

The results of this study showed a reduction in TBARS values in the FST group, and an increase in the CON group (Figure 2(d)), this difference also being significant (p = 0.001). This demonstrates a potential protective effect of FST against lipid peroxidation which subsequently results in damage to cellular membranes.

In regard to oxidative damage to DNA, 8-oxoDG levels decreased in both the FST and placebo groups (Figure 2(e)), but to a much greater degree in the FST group. This resulted in a significant difference between groups after the 6-week treatment (p < 0.001). Thus, FST may be considered a viable, natural supplementation that provides notable protection to DNA structure and integrity.

### 4.4. Growth-Related Factors

**Figure 3:**
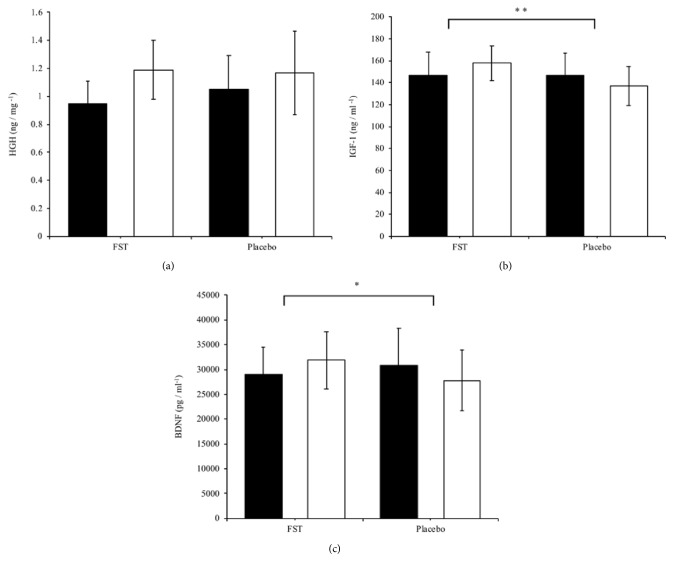
Changes in serum growth-related markers: (a) HGH, (b) IGF-1, and (c) BDNF levels were determined in the FST and placebo group, pre- and postexperimental trial. Each bar represents the mean ± SD. Student's paired* t*-tests were carried out to determine significant differences (*∗*p < 0.05, *∗∗*p < 0.001) between the FST and placebo groups.

The results on growth-related factors show no significant difference between groups in HGH levels (Figure 3(a)). A significant increase was observed in the FST group compared to the control group with respect to IGF-1 levels (p < 0.001) (Figure 3(b)). There was also a significant increase in BDNF serum levels as a result of FST supplementation, compared to the control group (Figure 3(c)) (p < 0.05).

## 5. Discussion

The search for exogenous sources of antioxidants has led to research into antioxidant nutrients (in this case, aquatic based food sources) to find accessible and biologically safe countermeasures to oxidative stress.* Laminaria japonica *A., an ingredient rich in vitamins, minerals, essential fatty acids, and bioactive compounds such as fucoidans, has been investigated for its numerous health preserving and enhancing effects, including its antioxidant properties. The current study demonstrated that FST supplementation for a 6-week period enhanced the activity of SOD, GPx, and GSR, while reducing biomarkers of lipid peroxidation (TBARS) and oxidative DNA damage (8-oxodG). These improvements in the antioxidant defense system coincided with the maintenance and/or improvement of neuropsychological test scores that are typically used to identify cases of MCI. In addition, 6MW and TUG test times were improved, along with increased serum IGF-1 and BDNF levels, indicating that FST may also act to preserve health-related fitness outcomes. These results have shed greater light on the antioxidant potential of FST and build on our recent study demonstrating a preservative effect of FST on cognitive ability. Unlike previous studies, this research has highlighted the apparent central and peripheral impact of bioactive properties in FST (GABA) and how they may interact to induce widespread prophylactic effects.

The cells' main defense mechanism against oxygen free radicals includes enzymes such as SOD, GSR, and GPx, among other low-molecular weight antioxidants and antioxidant nutrients. SOD is a key enzyme that converts superoxide (O_2_^−^) to H_2_O_2_. The superoxide anion is generated by numerous pathways [45] and, though not being considered the most harmful ROS, can reduce transition metals (e.g., Fe^+3^ to Fe^+2^), which are bound to biological sites, for use in a Fenton-type reaction, subsequently yielding highly toxic hydroxyl radicals. O_2_^−^ also readily reacts with nitric oxide (NO^∙^) to form peroxynitrite (ONOO^−^), a highly reactive substance that is also implicated in DNA damage [46]. Lee et al. [23] reported that FST quenched O_2_^−^ radicals, while Kang et al. [28] went on to demonstrate a significant augmentation in SOD activity following 4 weeks of FST supplementation. It is of particular importance that the present study corroborated these results in senior subjects, whose aging cells grow increasingly inefficient at eliminating oxidative stressors, seen particularly in the mitochondrion, where there are increased ROS production and even damaged membrane integrity, with age [47]. The scavenging of O_2_^−^ by SOD will prevent its involvement in the Haber-Weiss reaction and thus reduce OH^*∙*^ production. This in turn will reduce the potential for site-specific biological damage to lipid membranes, proteins, and DNA [48].

Though there is a reactive byproduct of superoxide dismutation, in the form of H_2_O_2_, it may be effectively detoxified to water by GPx in a reduction reaction involving glutathione (GSH) [49]. GSH, an abundant, low-molecular weight antioxidant, is a vital component of the cellular antioxidant system, and among its many important roles, it is responsible for scavenging highly reactive ROS (e.g., peroxynitrite, lipid peroxyl radical, and H_2_O_2_). The involvement of GPx in the decomposition of H_2_O_2_ is essential in preventing direct and/or indirect deleterious chemical effects such as oxidation of DNA, lipids, and –SH groups or sourcing the production of more deleterious species, namely, OH^*∙*^, respectively. Furthermore, reduced GPx activity has been identified as a predictor of increased cardiovascular risk [50, 51]. The administration of FST led to the enhanced activity of GPx after 6 weeks of supplementation, despite a 4-week study by Kang et al. [28] showing no significant effect of FST ingestion on GPx activity. Discrepancies between studies may be due to decreased GPx activity in the senior cohort used in the present study, thus allowing the protective effects of FST to be more significant compared to the placebo group. Nonetheless, our results build on previous findings showing FST to attenuate oxidative stress in rats with ethanol-induced hepatotoxicity [52] by demonstrating its potency in human subjects. Tight regulation of GSH, and its oxidized form (GSSG), is imperative to cell survival so much so that an imbalance in GSH has been linked to a wide range of neurodegenerative conditions, including MCI [18, 53], Alzheimer's disease, and aging [54]. GSR works hand-in-hand with GPx to help regulate the redox state of GSH (GSH:GSSG ratio) for many functions, including decomposition of H_2_O_2_. This makes the observation that GSR reduces during the latter stages of life [55], a particular concern to the aging population. Our study demonstrated a significant difference in GSR activity between the FST and CON group. Therefore, considering that GSH is a vital scavenger, recycled by GSR to combat oxidative stress, it follows that an upregulation of GSR and GPx via dietary intervention would aid in optimizing its redox state and thus reduce deleterious oxidative damage, related to MCI.

Targets especially susceptible to oxidation include cellular membranes, due to their high concentrations of polyunsaturated fatty acids [56]. We measured the levels of thiobarbituric reactive species, the end-products of whole-organism lipid peroxidation. Consistent with previous findings [21, 22, 29, 57], FST ingestion had a significant ameliorative effect on lipid peroxidation, lowering TBARS levels. Lipid peroxidation has been extensively studied and found to be significantly increased in the central nervous system and peripheral tissues of patients with AD and MCI [58, 59]. If the deleterious modification of cellular membranes can be alleviated by dietary supplementation, FST may provide a viable defense against lipid-peroxidation-associated neurodegenerative disease [45, 60]. DNA, another vulnerable site of oxidative damage, was yet to be reported on with regard to the antioxidant effects of FST. Thus, the present study recorded the changes in the DNA-oxidized adduct, 8-oxoDG, following FST supplementation. A significant protective effect against free radical-induced DNA damage was observed in the FST group, compared to the CON. This finding is important, as 8-oxoDG, one of the predominant forms of free radical-induced oxidative lesion, is widely considered a critical biomarker of oxidative stress [61], utilized in identifying both nuclear (nDNA) and mtDNA damage, associated with aging [62]. It is proposed that accumulation of DNA oxidative modifications in neurons may lead to inhibition of transcription factor binding that could lead to diminished transcription of critical antioxidant enzymes [9, 11]. Therefore, FST may be considered an effective and versatile dietary source of antioxidants, capable of providing DNA protection, particularly antimutagenic effects [26].

A clinical indicator of transition between normal aging and early dementia is MCI and presents as the most opportunistic phase for intervention. A practical clinical assessment of cognitive status, in the K-MMSE, was implemented to ascertain the impact of FST on subjects' orientation to time and place, recall ability, short-term memory, and arithmetic ability [39]. Additional cognitive test instruments were used to evaluate a wider spectrum of cognitive processes including, visual sensory memory (Iconic memory test), visual and spatial reasoning (Raven's test), selective attention (Flanker test), visual-conceptual and visual-motor ability (TMT), and numerical memory. Our results indicate a possible preservative and even enhancing effect of FST on cognitive ability in the elderly. We have recently investigated the effectiveness of FST at reversing scopolamine-induced dementia and alcohol-related amnesia and dementia and found significant improvements in hippocampus-dependent spatial learning ability and short-term memory. In addition to alcohol-induced MCI [63, 64], elevated OxS is also known to be present in age-related late-stage Alzheimer's disease and vulnerable regions of the MCI brain [9]. There is growing evidence supporting increased antioxidant capacity as the prime underlying mechanism by which FST acts to offset age-related degeneration. Studies have reported reduced diphenylpicrylhydrazyl (DPPH) levels, xanthine oxidase inhibition [21], increased superoxide radical scavenging, enhanced catalase, decreased malondialdehyde (MDA) levels [22], and decreased lipid peroxidation [29] following FST supplementation. Thus, by reporting on improvements in neuropsychological and physical function assessments, the current study builds on these previous findings demonstrating a possible relationship between improvements in antioxidant capacity and the preservation of both physical and cognitive capacity, in the aging population.

GABA is believed to play a key role in the antioxidant effects of FST, which may provide a range of health-promoting benefits in MCI. GABA administration has been shown to inhibit the formation of advanced lipoxidation end-products [30] and, more recently, decrease MDA concentration while increasing the activities of superoxide dismutase (SOD) and glutathione peroxidase (GPx) in the cerebral cortex and hippocampus [31], brain regions crucial to memory formation. The present study also corroborates our previous findings indicating a relationship between GABA-enriched FST intake and an increase in muscle-related growth factors (human growth hormone, insulin-like growth factor-1) and increase serum brain-derived neurotrophic factor (BDNF) [32], known to provide protection for innervating motor neurons, stimulate protein synthesis [33, 34], and maintain neuromuscular integrity [32] in age-related muscular degeneration [35]. However, it is crucial to add that blood-brain barrier permeability to GABA is a controversial topic. Nonetheless, preliminary evidence that GABA intake might help improve specific cognitive outcomes including temporal attention [65] may support the school of thought that describes a dynamic BBB through which some passage of solutes can occur by transcytosis, carrier-mediated transport, or simple diffusion of hydrophobic substances [66, 67]. Thus, it is reasonable to suggest a link between GABA ingestion and the aforementioned positive central effects and neuropsychological improvements. Improvements in TUG and 6MW tests may be an indication of BDNF-induced preservation of neuromuscular function as a result of FST supplementation. BDNF is also strongly implicated in the pathogenesis of dementia [36], AD [37], and Parkinson's disease [38]. Despite the lack of mechanistic investigations on how FST elicits scavenging effects on biologically harmful oxidants, it has been put forward that the bioactive properties of FST are found in the membrane complex, where polysaccharides containing a high percentage of L-fucose and sulfated ester groups exist. Of these, fucoidans have been extensively studied for their diverse biological activities, including antiobesity [68], anti-inflammation [69], and anticoagulant [70]. Parallels may be drawn between the antioxidant effects of fucoidan and those observed when administering FST, notably, superoxide scavenging [24, 71], inhibition of hydrogen peroxide-induced hemolysis [72], and prevention of increased lipid peroxides [68]. The antioxidant potency of fucoidan is said to be related to its molecular weight and sulfate-to-fucose ratio [73]. However, more research is required to ascertain the extent of fucoidan contribution and optimal dosages, to maximize effectiveness and avoid any negative side effects (e.g., fucoidan toxicity) [74].

## 6. Conclusion

The present study highlights the potential widespread application of FST in protecting against the degenerative effects of aging on short-term memory and physical function. Neuropsychological evaluation indicates that FST may provide a protective mechanism against cognitive impairment associated with dementia. In addition, neuromuscular integrity and physical function are typically compromised in aging and dementia patients; thus, whether by stimulation of muscle-related growth factors or an increase in serum BDNF, FST supplementation may act to preserve physical function in the elderly. Improvements in antioxidant activity following FST supplementation may indicate that the bioactivity of GABA or fucoidans act to provide a defense against progressive degeneration purportedly caused by reactive oxygen species. The fermentation of* Laminaria japonica* A. producing GABA-enriched FST seems to be a key factor in increasing the bioavailability of its main bioactive constituents and hence its antioxidant potency. Not only is this particular type of sea tangle readily available and widely consumed across Asia, but there are a growing number of in vivo studies demonstrating the diverse health benefits of fermented edible seaweeds [75]. In addition, the environmentally friendly fermentation process removes any odor making the supplementation of FST more palatable, which is key in a more environmentally aware society where there is growing demand for natural foods.

## Figures and Tables

**Table 1:** Participant characteristics.

	Placebo (n=32)	Treatment (n=28)
Age (years)	74.57 ± 5.69	72.35 ± 5.54
Height (cm)	153.47 ± 6.68	157.70 ± 5.84
Weight (kg)	54.20 ± 7.35	59.30 ± 6.93

**Table 2:** Culture broth composition.

	**Sea tangle**	**Water**	**Yeast extract**	**Glucose**	**Seed culture**	**Total**
Weight (g)	408.5	6,127.5	10	5	76.5	6,627.5
Percentage (%)	6.2	92.5	0.2	0.1	1.2	100

*γ*-aminobutyric acid-enriched fermented sea tangle  :  water = 1 : 15 (w/v). Reprinted from “effects of *γ*-aminobutyric acid-enriched fermented sea tangle (Laminaria japonica) on brain derived neurotrophic factor-related muscle growth and lipolysis in middle aged women,”* *Algae, vol. 31, no. 2, pp. 177, 2016. Reprinted with permission.

**Table 3:** Pre and posttest comparison results of neuropsychological and physical fitness assessment using t-test analysis, between FST group and placebo group.

		FST (*n=32*)		Placebo (*n=28*)		*t*	*P*
		*M(SD)*	Difference *M(SD)*	*M(SD)*	Difference *M(SD)*		
K-MMSE	pre	26.31(1.78)	1.59(1.49)	26.35(2.79)	.39(1.85)	2.773	.007
	post	27.90(1.57)		26.75(3.52)			

Numerical memory test	pre	7.78(.42)	.62(.90)	7.92(.85)	.10(.99)	2.110	.039
	post	8.40(.87)		8.03(1.23)			

Raven's test	pre	11.06(5.29)	2.37(3.63)	12.25(5.10)	−1.67(2.85)	4.754	.000
	post	13.43(6.17)		10.57(4.51)			

Flanker test	pre	1785.95(385.77)	−549.18 (947.68)	1785.36(457.92)	−610.36 (950.78)	.247	.806
	post	1068.92(279.10)		1036.27(344.85)			

Iconic memory test (pre)	pre	.45(.20)	.15(.23)	.48(.22)	.05(.22)	1.606	.114
	post	.61(.13)		.54(.17)			

Iconic memory test (simultaneous)	pre	.22(.13)	.16(.12)	.25(.11)	.05(.10)	3.673	.001
	post	.39(.10)		.31(.14)			

Iconic memory test (post)	pre	.15(.09)	.10(.11)	.18(.09)	.018(.12)	2.660	.010
	post	.26(.11)		.20(.10)			

TMT	pre	66.99(29.94)	−5.39(15.48)	77.38(34.37)	−10.98(26.13)	1.024	.310
	post	61.60(20.43)		65.98(32.96)			

6MW (1)	pre	4.81(.61)	−.32(.46)	4.64(.75)	.26(.40)	−5.232	.000
	post	4.48(.43)		4.90(.66)			

6MW (2)	pre	4.65(.93)	−.11(.55)	4.53(.83)	.38(.57)	−3.382	.001
	post	4.54(.66)		4.91(.81)			

TUG (1)	pre	6.96(.96)	−.14(1.35)	6.59(.90)	1.25(.75)	−4.861	.000
	post	6.82(.73)		7.84(1.27)			

TUG (2)	pre	7.01(.99)	−.29(1.17)	6.60(.97)	1.45(1.31)	1.063	.292
	post	6.71(.70)		8.05(1.75)			

## Data Availability

The data used to support the findings of this study are available from the corresponding author upon request.

## References

[1] Giebel C. M., Sutcliffe C., Challis D. (2015). Activities of daily living and quality of life across different stages of dementia: A UK study. *Aging & Mental Health*.

[2] Alzheimer's Association (2016). *Alzheimer's disease facts and figures*.

[3] Prince M., Bryce R., Albanese E., Wimo A., Ribeiro W., Ferri C. P. (2013). The global prevalence of dementia: a systematic review and metaanalysis. *Alzheimer’s & Dementia*.

[4] Praticò D., Clark C. M., Liun F., Lee V. Y. M., Trojanowski J. Q. (2002). Increase of brain oxidative stress in mild cognitive impairment: a possible predictor of Alzheimer disease. *JAMA Neurology*.

[5] Zhao Y., Zhao B. (2013). Oxidative stress and the pathogenesis of Alzheimer's disease. *Oxidative Medicine and Cellular Longevity*.

[6] Santos J. R., Gois A. M., Mendonça D. M., Freire M. A. (2014). *in Alzheimers disease*.

[7] Cervellati C., Romani A., Seripa D. (2014). Systemic oxidative stress and conversion to dementia of elderly patients with mild cognitive impairment. *BioMed Research International*.

[8] Aliev G., Priyadarshini M., Reddy V. P. (2014). Oxidative stress mediated mitochondrial and vascular lesions as markers in the pathogenesis of alzheimer disease. *Current Medicinal Chemistry*.

[9] Lovell M. A., Markesbery W. R. (2007). Oxidative DNA damage in mild cognitive impairment and late-stage Alzheimer's disease. *Nucleic Acids Research*.

[10] Price J. L., McKeel D. W., Buckles V. D. (2009). Neuropathology of nondemented aging: presumptive evidence for preclinical Alzheimer disease. *Neurobiology of Aging*.

[11] Wang X., Wang W., Li L., Perry G., Lee H. G., Zhu X. (2013). Oxidative stress and mitochondrial dysfunction in Alzheimer's disease. *Biochimica et Biophysica Acta*.

[12] Petersen R. C. (2004). Mild cognitive impairment as a diagnostic entity. *Journal of Internal Medicine*.

[13] Nordahl C. W., Ranganath C., Yonelinas A. P., DeCarli C., Reed B. R., Jagust W. J. (2005). Different mechanisms of episodic memory failure in mild cognitive impairment. *Neuropsychologia*.

[14] Du A. T., Schuff N., Amend D. (2001). Magnetic resonance imaging of the entorhinal cortex and hippocampus in mild cognitive impairment and Alzheimer's disease , Journal of Neurology. *Neurosurgery Psychiatry*.

[15] Lu Z.-L., Neuse J., Madigan S., Dosher B. A. (2005). Fast decay of iconic memory in observers with mild cognitive impairments. *Proceedings of the National Acadamy of Sciences of the United States of America*.

[16] Ambra F. I., Iavarone A., Ronga B. (2016). Qualitative patterns at Raven’s colored progressive matrices in mild cognitive impairment and Alzheimer’s disease. *Aging Clinical and Experimental Research*.

[17] Praticò D. (2008). Oxidative stress hypothesis in Alzheimer's disease: a reappraisal. *Trends in Pharmacological Sciences*.

[18] Rinaldi P., Polidori M. C., Metastasio A. (2003). Plasma antioxidants are similarly depleted in mild cognitive impairment and in Alzheimer's disease. *Neurobiology of Aging*.

[19] Aslam A., Misbah S. A., Talbot K., Chapel H. (2004). Vitamin E deficiency induced neurological disease in common variable immunodeficiency: Two cases and a review of the literature of vitamin E deficiency. *Clinical Immunology*.

[20] Howard L., Ovesen L., Satya Murti S., Chu R. (1982). Reversible neurological symptoms caused by vitamin E deficiency in a patient with short bowel syndrome. *American Journal of Clinical Nutrition*.

[21] Lee B.-J., Senevirathne M., Kim J.-S. (2010). Protective effect of fermented sea tangle against ethanol and carbon tetrachloride-induced hepatic damage in Sprague-Dawley rats. *Food and Chemical Toxicology*.

[22] Kang Y.-M., Qian Z.-J., Lee B.-J., Kim Y.-M. (2011). Protective effect of GABA-enriched fermented sea tangle against ethanol-induced cytotoxicity in HepG2 cells. *Biotechnology and Bioprocess Engineering*.

[23] Lee B.-J., Kim J.-S., Kang Y. M. (2010). Antioxidant activity and *γ*-aminobutyric acid (GABA) content in sea tangle fermented by Lactobacillus brevis BJ20 isolated from traditional fermented foods. *Food Chemistry*.

[24] Wang J., Zhang Q., Zhang Z., Li Z. (2008). Antioxidant activity of sulfated polysaccharide fractions extracted from *Laminaria japonica*. *International Journal of Biological Macromolecules*.

[25] Chiu K. W., Fung A. Y. L. (1997). The cardiovascular effects of green beans (Phaseolus aureus), common rue (Ruta graveolens), and kelp (Laminaria japonica) in rats. *General Pharmacology: The Vascular System*.

[26] Okai Y., Higashi-Okai K., Nakamura S.-I. (1993). Identification of heterogenous antimutagenic activities in the extract of edible brown seaweeds, Laminaria japonica (Makonbu) and Undaria pinnatifida (Wakame) by the umu gene expression system in Salmonella typhimurium (TA1535/pSK1002). *Mutation Research Letters*.

[27] Lee S., Kim C., Jang H., Cho S., Choi J. (2011). Anti-hyperlipidemia and Anti-arteriosclerosis Effects of Laminaria japonica in Sprague-Dawley Rats. *Fisheries and Aquatic Sciences*.

[28] Kang Y. M., Lee B.-J., Kim J. I. (2012). Antioxidant effects of fermented sea tangle (Laminaria japonica) by Lactobacillus brevis BJ20 in individuals with high level of *γ*-GT: A randomized, double-blind, and placebo-controlled clinical study. *Food and Chemical Toxicology*.

[29] Cha J.-Y., Lee B.-J., Je J.-Y., Kang Y.-M., Kim Y.-M., Cho Y.-S. (2011). GABA-enriched fermented Laminaria japonica protects against alcoholic hepatotoxicity in Sprague-Dawley rats. *Fisheries and Aquatic Sciences*.

[30] Deng Y., Xu L., Zeng X., Li Z., Qin B., He N. (2010). New perspective of GABA as an inhibitor of formation of advanced lipoxidation end-products: It's interaction with malondiadehyde. *Journal of Biomedical Nanotechnology*.

[31] Deng Y., Wang W., Yu P. (2013). Comparison of taurine, GABA, Glu, and Asp as scavengers of malondialdehyde in vitro and in vivo. *Nanoscale Research Letters*.

[32] Choi W.-C., Reid S. N. S., Ryu J.-K., Kim Y., Jo Y.-H., Jeon B. H. (2016). Effects of *γ*-aminobutyric acid-enriched fermented sea tangle (Laminaria japonica) on brain derived neurotrophic factor-related muscle growth and lipolysis in middle aged women. *Algae*.

[33] Oppenheim R. W., Qin-Wei Y., Prevette D., Yan Q. (1992). Brain-derived neurotrophic factor rescues developing avian motoneurons from cell death. *Nature*.

[34] Zhang X.-H., Poo M.-M. (2002). Localized synaptic potentiation by BDNF requires local protein synthesis in the developing axon. *Neuron*.

[35] Cruz-Jentoft A. J., Baeyens J. P., Bauer J. M. (2010). Sarcopenia: European consensus on definition and diagnosis. *Age and Ageing*.

[36] Lee J. G., Shin B. S., You Y. S. (2009). Decreased serum brain-derived neurotrophic factor levels in elderly Korean with dementia. *Psychiatry Investigation*.

[37] Laske C., Stransky E., Leyhe T. (2007). BDNF serum and CSF concentrations in Alzheimer’s disease, normal pressure hydrocephalus and healthy controls. *Journal of Psychiatric Research*.

[38] Scalzo P., Kümmer A., Bretas T. L., Cardoso F., Teixeira A. L. (2010). Serum levels of brain-derived neurotrophic factor correlate with motor impairment in Parkinson's disease. *Journal of Neurology*.

[39] Folstein M. F., Folstein S. E., McHugh P. R. (1975). “Mini mental state”. A practical method for grading the cognitive state of patients for the clinician. *Journal of Psychiatric Research*.

[40] Jones R. N., Gallo J. J. (2000). Dimensions of the Mini-Mental State Examination among community dwelling older adults. *Psychological Medicine*.

[41] Raven J., Raven J. C., Court J. H. (2000). *Raven manual: Section 3, standard progressive matrices, including the parallel and plus versions*.

[42] Salthouse T. A., Fristoe N. M. (1995). Process analysis of adult age effects on a computer-administered trail making test. *Neuropsychology*.

[43] Lee K.-B., Lee P., Yoo S.-W., Kim Y.-D. (2016). Reliability and validity of the Korean version of the community balance and mobility scale in patients with hemiplegia after stroke. *Journal of Physical Therapy Science*.

[44] Steffen T. M., Hacker T. A., Mollinger L. (2002). Age- and gender-related test performance in community-dwelling elderly people: six-minute walk test, Berg balance scale, timed up & go test, and gait speeds. *Physical Therapy in Sport*.

[45] Fang Y.-Z., Yang S., Wu G. (2002). Free radicals, antioxidants, and nutrition. *Nutrition Journal *.

[46] Kujoth G. C., Hiona A., Pugh T. D. (2005). Medicine: mitochondrial DNA mutations, oxidative stress, and apoptosis in mammalian aging. *Science*.

[47] Brunk U. T., Terman A. (2002). The mitochondrial-lysosomal axis theory of aging: accumulation of damaged mitochondria as a result of imperfect autophagocytosis. *European Journal of Biochemistry*.

[48] Kawanishi S., Hiraku Y., Murata M., Oikawa S. (2002). The role of metals in site-specific DNA damage with reference to carcinogenesis. *Free Radical Biology & Medicine*.

[49] Chance B., Sies H., Boveris A. (1979). Hydroperoxide metabolism in mammalian organs. *Physiological Reviews*.

[50] Buijsse B., Lee D.-H., Steffen L. (2012). Low serum glutathione peroxidase activity is associated with increased cardiovascular mortality in individuals with low HDLc's. *PLoS ONE*.

[51] Holley A. S., Harding S. A., Sasse A., Miller J. H., Larsen P. D. (2016). Reduced glutathione peroxidase activity predicts increased cardiovascular risk following an acute coronary syndrome.. *International Cardiovascular Forum Journal*.

[52] Cha J.-Y., Senevirathne M., Lee B.-J. (2013). Fermented Sea Tangle (Laminaria Japonica) Attenuates Ethanol-Induced Oxidative Stress In Sprague-Dawley Rats. *Journal of Food Biochemistry*.

[53] Torres L. L., Quaglio N. B., de Souza G. T. (2011). Peripheral oxidative stress biomarkers in mild cognitive impairment and alzheimer's disease. *Journal of Alzheimer's Disease*.

[54] Liu H., Wang H., Shenvi S., Hagen T. M., Liu R.-M. (2014). Glutathione metabolism during aging and in Alzheimer disease. *Annals of the New York Academy of Sciences*.

[55] Sohal R. S., Arnold L., Orr W. C. (1990). Effect of age on superoxide dismutase, catalase, glutathione reductase, inorganic peroxides, TBA-reactive material, GSH/GSSG, NADPH/NADP+ and NADH/NAD+ in Drosophila melanogaster. *Mechanisms of Ageing and Development*.

[56] Halliwell B., Gutteridge J. M. (1999). *Free Radical Biology & Medicine*.

[57] Park M.-J., Han J.-S. (2013). Protective effects of the fermented laminaria japonica extract on oxidative damage in LLC-PK1 cells. *Preventive Nutrition and Food Science*.

[58] Baldeiras I., Santana I., Proença M. T. (2008). Peripheral oxidative damage in mild cognitive impairment and mild Alzheimer's disease. *Journal of Alzheimer's Disease*.

[59] Greilberger J., Koidl C., Greilberger M. (2008). Malondialdehyde, carbonyl proteins and albumin-disulphide as useful oxidative markers in mild cognitive impairment and Alzheimer's disease. *Free Radical Research*.

[60] Shichiri M. (2014). The role of lipid peroxidation in neurological disorders. *Journal of Clinical Biochemistry and Nutrition*.

[61] Valavanidis A., Vlachogianni T., Fiotakis C. (2009). 8-Hydroxy-2′ -deoxyguanosine (8-OHdG): a critical biomarker of oxidative stress and carcinogenesis. *Journal of Environmental Science and Health, Part C: Environmental Carcinogenesis and Ecotoxicology Reviews*.

[62] Hamilton M. L., van Remmen H., Drake J. A. (2009). Does oxidative damage to DNA increase with age?. *Proceedings of the National Acadamy of Sciences of the United States of America*.

[63] Gupta S., Warner J. (2008). Alcohol-related dementia: a 21st-century silent epidemic?. *The British Journal of Psychiatry*.

[64] Ridley N. J., Draper B., Withall A. (2013). Alcohol-related dementia: An update of the evidence. *Alzheimer’s Research & Therapy*.

[65] Leonte A., Colzato L. S., Steenbergen L., Hommel B., Akyürek E. G. (2018). Supplementation of gamma-aminobutyric acid (GABA) affects temporal, but not spatial visual attention. *Brain and Cognition*.

[66] Shyamaladevi N., Jayakumar A. R., Sujatha R., Paul V., Subramanian E. H. (2002). Evidence that nitric oxide production increases *γ*-amino butyric acid permeability of blood-brain barrier. *Brain Research Bulletin*.

[67] Steenbergen L., Sellaro R., Stock A.-K., Beste C., Colzato L. S. (2015). *γ*-Aminobutyric acid (GABA) administration improves action selection processes: A randomised controlled trial. *Scientific Reports*.

[68] Park M.-K., Jung U., Roh C. (2011). Fucoidan from marine brown algae inhibits lipid accumulation. *Marine Drugs*.

[69] Kang S.-M., Kim K.-N., Lee S.-H. (2011). Anti-inflammatory activity of polysaccharide purified from AMG-assistant extract of Ecklonia cava in LPS-stimulated RAW 264.7 macrophages. *Carbohydrate Polymers*.

[70] Athukorala Y., Jung W.-K., Vasanthan T., Jeon Y.-J. (2006). An anticoagulative polysaccharide from an enzymatic hydrolysate of Ecklonia cava. *Carbohydrate Polymers*.

[71] Li D. Y., Xu R. Y., Zhou W. Z. (2002). Effects of fucoidan extracted from brown seaweed on lipid peroxidation in mice. *Acta Nutrimenta Sinica*.

[72] Zhang Q., Yu P., Zhou P. (2003). Studies on antioxidant activities of fucoidan from Laminaria japonica. *Chinese traditional and herbal Drugs*.

[73] Xue C.-H., Fang Y., Lin H. (2001). Chemical characters and antioxidative properties of sulfated polysaccharides from Laminaria japonica. *Journal of Applied Phycology*.

[74] Li N., Zhang Q., Song J. (2005). Toxicological evaluation of fucoidan extracted from *Laminaria japonica* in Wistar rats. *Food and Chemical Toxicology*.

[75] Chye F. Y., Ooi P. W., Ng S. Y., Sulaiman M. R. (2018). Fermentation-Derived Bioactive Components from Seaweeds: Functional Properties and Potential Applications. *Journal of Aquatic Food Product Technology*.

