# Corrigendum to “The Effects of Fermented* Laminaria japonica* on Short-Term Working Memory and Physical Fitness in the Elderly”

**DOI:** 10.1155/2018/1764038

**Published:** 2018-09-09

**Authors:** Storm N. S. Reid, Je-kwang Ryu, Yunsook Kim, Byeong Hwan Jeon

**Affiliations:** ^1^Department of Physical Education, School of Sports and Health, Kyungsung University, Busan 48434, Republic of Korea; ^2^Institute for Cognitive Science, College of Humanities, Seoul National University, Seoul 08826, Republic of Korea; ^3^Marine Bio-Industry Development Center, Marine Bioprocesses Co., Ltd., Busan 46048, Republic of Korea

In the article titled “The Effects of Fermented* Laminaria japonica* on Short-Term Working Memory and Physical Fitness in the Elderly” [1], the mention of the implementation of a “6-minute walk test” was inaccurate, where it should be “6-meter walk test”. Therefore, “6-minute walk test” should be corrected to “6-meter walk test” in the Abstract and the Material and Methods. Additionally, the reference Helen S. P. Lam et al. [2] should be added as reference [76] in the original article.

The corrections should be applied as follows:

(i) In the Abstract, “Shorter test trial times in the 6-minute walk test were observed in the FST group (p<0.001 and p<0.05, trials 1 and 2, respectively)” should be corrected to “Shorter test trial times in the 6-meter walk test were observed in the FST group (p<0.001 and p<0.05, trials 1 and 2, respectively).”
(ii) In the Material and Methods, “6-Minute Walk Test (6MW). The 6MW test was used to measure the maximum distance that a person can walk in 6 minutes. This test is a submaximal aerobic capacity test that allows researchers to safely and simply assess physical function with the elderly and special populations [43, 44]” should be corrected to “6- Meter Walk Test (6MW). The 6MW test was used to measure the time it takes for a subject to walk a 6-meter straight line path. This test is a submaximal aerobic capacity test that allows researchers to safely and simply assess physical function with the elderly and special populations [76].”

## References

[1] Reid S. N. S., Ryu J. K., Kim Y., Jeon B. H. (2018). The effects of fermented* Laminaria japonica* on short-term working memory and physical fitness in the elderly. *Evidence-Based Complementary and Alternative Medicine*.

[2] Lam H. S. P., Lau F. W. K., Chan G. K. L., Sykes K. (2010). The validity and reliability of a 6-Metre Timed Walk for the functional assessment of patients with stroke. *Physiotherapy Theory and Practice*.

